# Testicular torsion with preserved flow: key sonographic features and value-added approach to diagnosis

**DOI:** 10.1007/s00247-018-4093-0

**Published:** 2018-02-21

**Authors:** Anjum N. Bandarkar, Anna R. Blask

**Affiliations:** 1 Department of Radiology, Mid-Atlantic Permanente Medical Group, 1890 Metro Center Drive, Reston, VA 20190 USA; 2 Department of Radiology, Children’s National Health System, Washington, DC USA

**Keywords:** Bell clapper anomaly, Children, Epididymis, Scrotum, Testis, Torsion, Ultrasound, Whirlpool sign

## Abstract

Testicular sonography has contributed greatly to the preoperative diagnosis of testicular torsion in the pediatric patient and is the mainstay for evaluation of acute scrotal pain. Despite its high sensitivity and specificity, both false-negative and false-positive findings occur. Presence of documented Doppler flow within the testis might be a dissuading factor for surgical exploration with resultant testicular loss in the false-negative cases. Our goal is to illustrate key sonographic features in the spectrum of testicular torsion with preserved testicular flow, and to describe how to differentiate testicular torsion from epididymitis in order to avoid the under-diagnosis of testicular torsion. We simplify the anatomy of the bell clapper testis. We also describe our sonographic protocol for testicular torsion and share valuable tips from our approach to challenging cases.

**Electronic supplementary material:**

The online version of this article (10.1007/s00247-018-4093-0) contains supplementary material, which is available to authorized users.

## Introduction

Recent investigators have emphasized the concept of intermittent and partial testicular torsion, which can be difficult to diagnose with sonography because these cases have either subtle decreased flow or flow that appears to be symmetrical with the contralateral testis, and symptoms can wax and wane [1]. Such cases might present diagnostic dilemmas for both the pediatric radiologist and urologist. Testicular torsion is not an all-or-none phenomenon and can be of complete, partial or intermittent types. The incidence of each type of torsion in isolation is unknown. In a study in which all children with symptoms of acute scrotum underwent surgical revision, torsion of the appendix testis was the most common pathology (57%), followed by torsion of the spermatic cord (27%) and much less commonly epididymitis (11%) [2].

Complete torsion occurs when the testis twists 360° or greater, usually leading to absence of intratesticular flow on color Doppler exam; however sometimes the flow is preserved or decreased. Intermittent torsion is defined as sudden onset of unilateral testicular pain of short duration with spontaneous resolution. In partial or incomplete torsion, the degree of spermatic cord twist is less than 360°, allowing for some residual perfusion to the testis. However there is no spontaneous resolution of pain [1].

Testicular torsion has been reported to have a bimodal distribution, an initial peak in the first year of age where the torsion is of extravaginal type, and a second surge in adolescence where intravaginal torsion is common. Cases of intravaginal torsion with preserved flow and their critical sonographic findings are described in this review. Preserved flow indicates these cases did not have completely absent flow (which is typically straightforward and diagnostic for testicular torsion) on color Doppler exam. Instead, the affected testis had either decreased or symmetrical preserved flow compared to the contralateral unaffected side, sometimes even increased flow [1]. In many of our cases, it was extremely difficult to decide whether the flow was truly symmetrical to the contralateral side or was subtly decreased. Of all the cases with preserved flow, decreased flow in the affected testis was more commonly observed than symmetrical or increased flow. The patient age in our cases ranged from 3 years to 19 years, the majority being 12 years and older. While studying some of our youngest patients (3–6 years), we noted that they all presented with abdominal pain, nausea and vomiting, and received the typical workup for pain including ultrasound examination to rule out appendicitis. The nonspecific symptoms resulted in a delayed diagnosis. It is important to remember that intravaginal torsion can occur even in such young patients, though less frequently compared to adolescents, and when presentation is right-sided it can mimic appendicitis. A clinical examination of the scrotum is recommended in all boys with right lower quadrant pain and a low threshold for a scrotal ultrasonography.

### Anatomy of the bell clapper testis

**Fig. 1:**
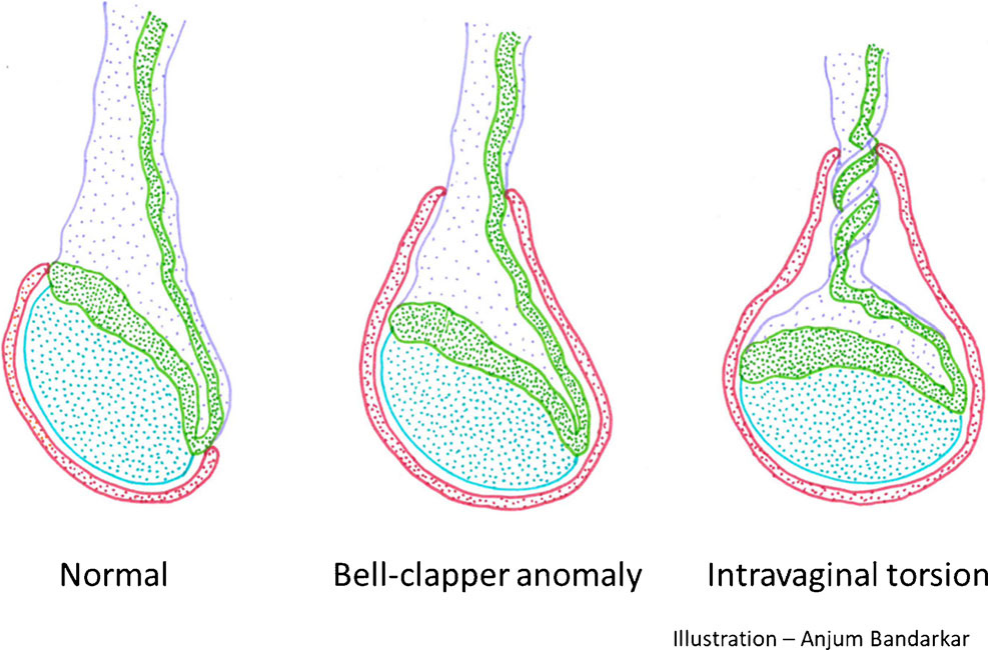
Anatomy of the normal testis, bell clapper anomaly and intravaginal testicular torsion. *Blue* testis, *Green* epididymis, *Lavender* spermatic cord and vessels, *Red* tunica vaginalis

The bell clapper anomaly has been defined as an abnormally high attachment of the tunica vaginalis parietal lamina to the spermatic cord such that the tunica vaginalis completely encircles the epididymis, the distal unattached spermatic cord and the testis rather than attaching to the posterolateral aspect of the testis (Fig. 1) [3].

Normally, the epididymis extends along the full length of the testis posterolaterally so that the upper and lower poles of the testis are covered and the tunica vaginalis parietal lamina is anchored to the epididymis. In a bell clapper anomaly, not only is there a lack of posterior attachment of the tunica vaginalis parietal lamina to the epididymis, but the epididymis–testis connection is also incomplete and the epididymis might be detached from the testis lower pole [4]. Depending on the degree of epididymal detachment, the entire unit comprising the testis, epididymis and distal spermatic cord hangs freely in the intravaginal space and can twist, resulting in the horizontal lie or testicular torsion. There is wide consensus that bell clapper anomaly is the primary risk factor for acute intravaginal torsion. An autopsy study revealed bell clapper anomaly in 12% of testes [5]. It was found to be bilateral in most cases. In a study of adolescent boys with acute testicular torsion, all torsion was intravaginal and was associated with ipsilateral bell clapper anomaly, and with contralateral bell clapper anomaly in 78% of the cases [4].

### Sonographic features most reliable for diagnosing testicular torsion

#### Spermatic cord whirlpool sign

**Fig. 2:**
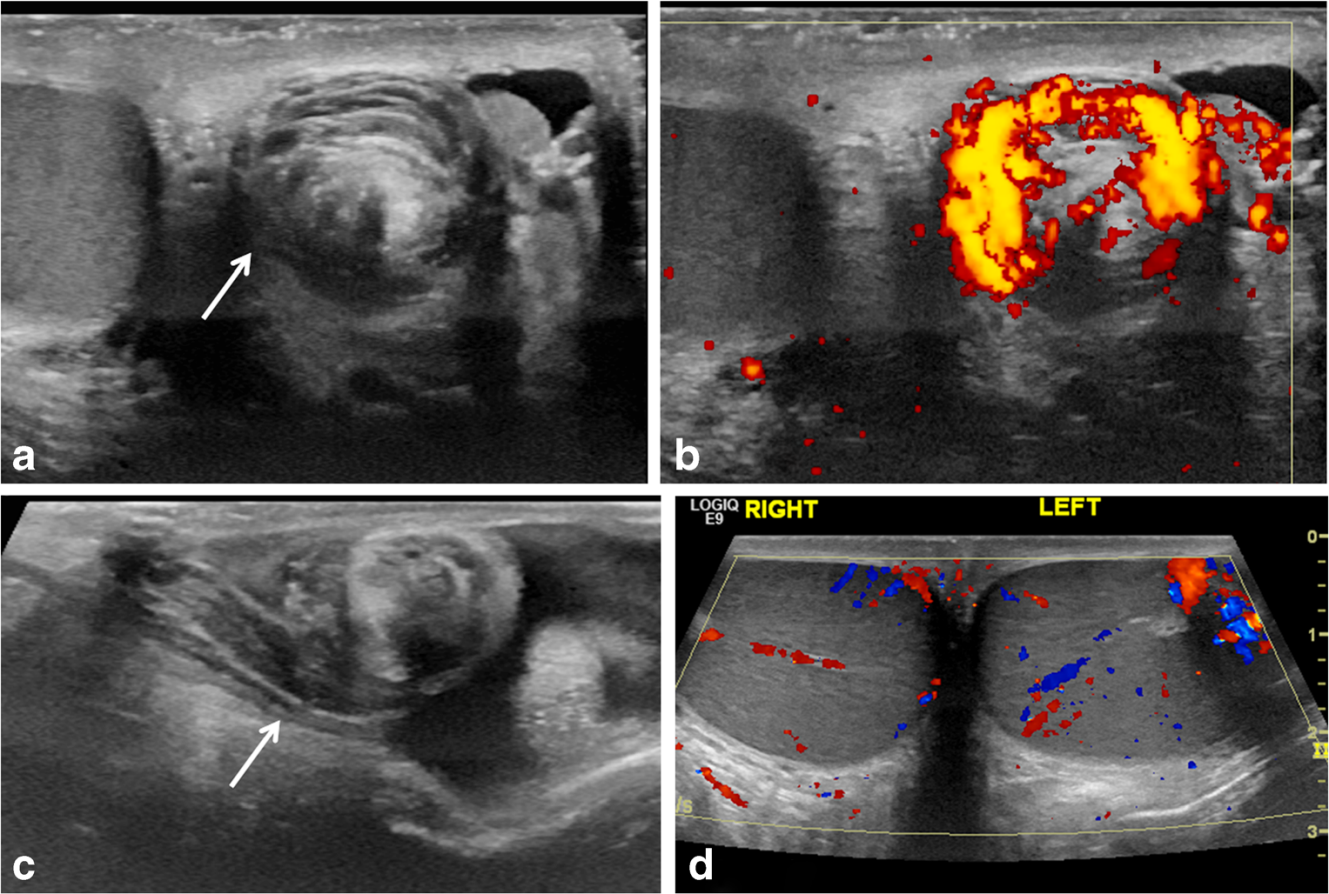
“Whirlpool sign” of spermatic cord. **a** Gray-scale transverse US image of upper left scrotal sac shows an eddy swirl (*arrow*) of the spermatic cord suggesting torsion of the cord. This 12-year-old boy woke with acute left testicular pain and experienced nausea and vomiting along with the pain. **b** Power Doppler US image of the same twisted cord shows concentric pattern of preserved flow in the vessels of the twisted cord. The flow in the left testis (not shown) was minimally decreased compared to the right side and bilateral bell clapper deformity was found during orchiopexy along with complete torsion of the left testis with 360° twist. **c** Gray-scale longitudinal US image of the left scrotum in a 13-year-old boy with 1 day of left-side pain shows abrupt spiral twisting of the spermatic cord (*arrow*) at the external inguinal ring, creating a whirlpool sign. **d** Color Doppler transverse image of the testes in the same boy as in (**c**) shows preserved and symmetrical flow bilaterally. After manual detorsion in the emergency room, he underwent orchiopexy and was diagnosed with intermittent torsion

The “whirlpool sign” is defined as an abrupt change in the course of the spermatic cord with a spiral twist at the external inguinal ring or in the scrotal sac (Fig. 2; videos 1, 2 and 3 in the online supplementary material). It is a reliable and direct sonographic sign that implies torsion of the spermatic cord and testis [6–8]. The classic whirlpool sign is observed less frequently compared to a tortuous redundant cord but is considered to be of great diagnostic significance [1].

#### Redundant spermatic cord

**Fig. 3:**
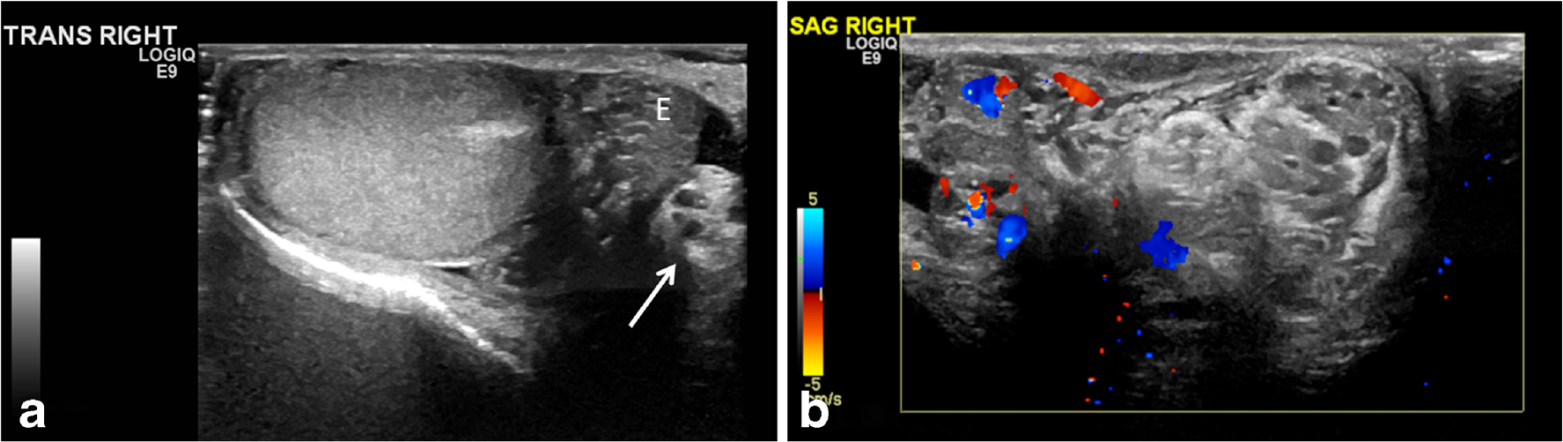
Intermittent torsion in a 17-year-old boy who presented with 5 h of acute right testicular pain after a game of football. He had experienced 6–7 similar episodes in the last 2 years where the pain had spontaneously resolved. Cremasteric reflex was absent on the right. **a** Gray-scale transverse US image of the right testis shows a redundant spermatic cord (*arrow*) occupying the medial half of the scrotal sac, with a mildly edematous epididymis (*E*) adjacent to it. The echogenic mediastinum testis faced medially instead of posterolaterally, which was concerning for altered testicular lie. **b** Color Doppler longitudinal image of the right scrotum shows excess and tortuous spermatic cord bunched up in the scrotal sac superior to the testis and formation of pseudomass, suggesting torsion of the spermatic cord. Note that this extratesticular pseudomass is not hyperemic and should not be confused with epididymitis. Orchiopexy was recommended; however the family chose to wait because his pain improved. Elective orchiopexy was performed 7 months later and bilateral bell clapper anomaly was noted; he was diagnosed with intermittent torsion

**Fig. 4:**
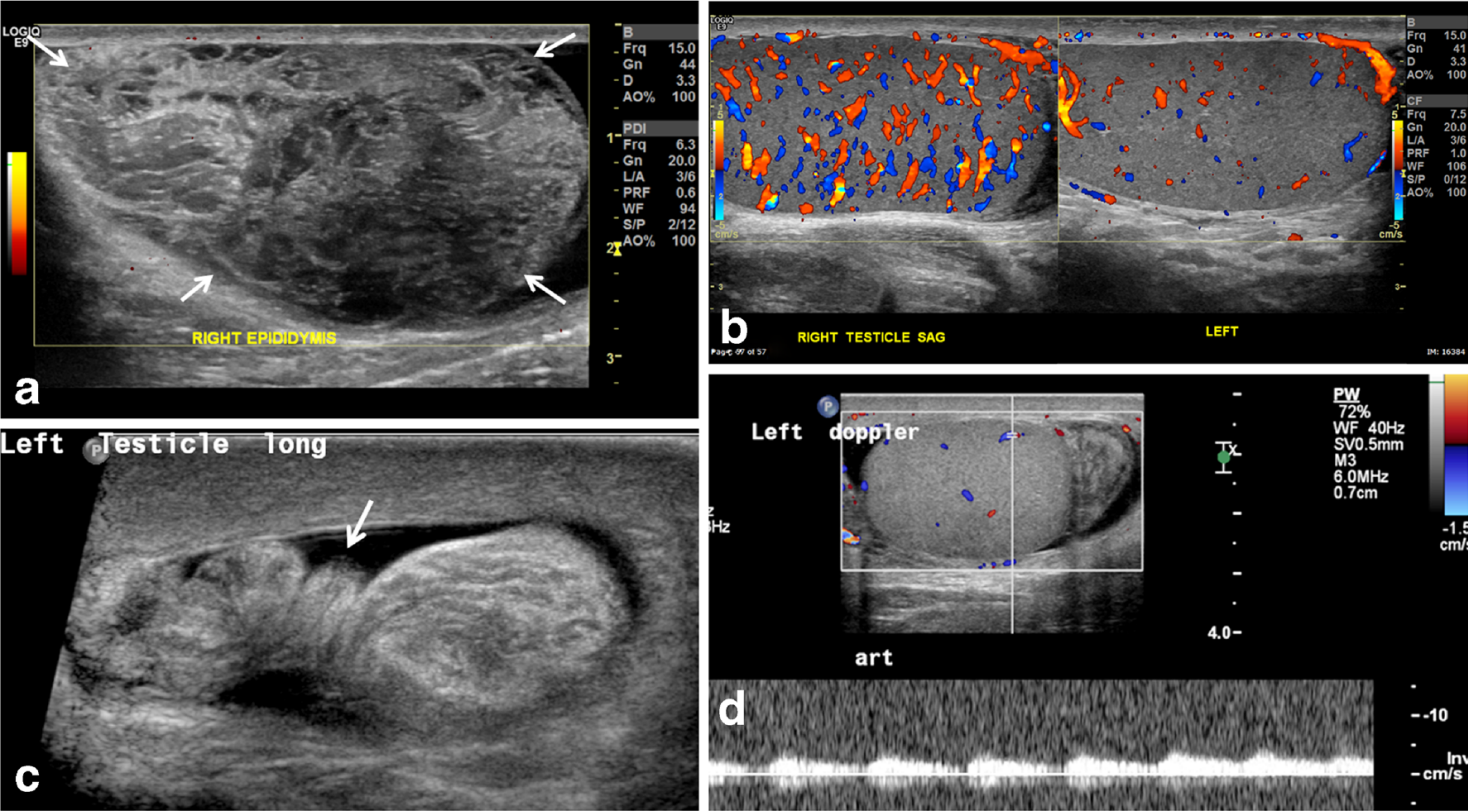
Redundant spermatic cord and enlarged epididymal-cord complex in the setting of preserved testicular flow. **a** Power Doppler US image in a 15-year-old boy who presented with acute right testicular pain shows an avascular pseudomass (*arrows*) along the superior aspect of the testis. **b** Scrotal color Doppler sonography shows preserved and increased testicular flow from manual detorsion but there is a bunched up cord as seen in Fig 4a, forming an enlarged epididymal-cord complex or extratesticular pseudomass. This suggests that the cord was tangled with and inseparable from the epididymis, with the point of twist forming the torsion knot. **c** Gray-scale US image at the level of the external inguinal ring in a 12-year-old boy with 24 h of acute left testicular pain shows a twisted redundant cord (*arrow*) in the lower inguinal canal (identified retrospectively) with a small surrounding hydrocele. **d** Color and pulsed wave Doppler US in the same boy as in (**c**) shows preserved left testicular flow. Because of the long duration of pain, which had begun to improve at the time of exam, and presence of intratesticular flow, he was given a diagnosis of epididymitis. He returned a week later with an infarcted left testis that showed ultrasound findings of late torsion. This example reiterates that duration of pain and presence of intratesticular flow should not deter one from looking for the twisted cord because the latter can clinch the diagnosis

Redundant spermatic cord can be described as the presence of excess and tortuous spermatic cord in the scrotal sac and is a very helpful sign of anomalous attachment of the tunica vaginalis. Normally, there should be no free piece of cord in the scrotal sac. Incidentally noted redundant spermatic cord, in the absence of acute pain, can be seen as a slightly wavy cord structure near the external ring. However in the setting of acute testicular pain, the redundant cord is usually seen bunched up and lying freely at the superior aspect of the testis or in the scrotal sac, surrounded by a small hydrocele that is typically reactive to the ongoing twisting and edema (Fig. 3; Video 4, online supplementary material). The bunched up cord often looks like an extratesticular, ovoid heterogeneous-echotexture mass that has been described as “boggy pseudomass,” typically seen below the point of torsion [1, 7] (Video 5, online supplementary material). The exact point of twisting of the cord is frequently indiscernible and hence the term “torsion knot” might be used interchangeably with boggy pseudomass, both implying a tangle of varying proportions of convolutions of the swollen spermatic cord with or without the epididymis [1]. It is crucial to put color on the presumed torsion knot (Fig. 4). It should not be mistaken for a paratesticular neoplasm, which most commonly presents as an asymptomatic painless mass. Presence of radiating, dilated anechoic tubules, which represent congested vessels, helps in recognizing cord structure in the torsion knot or pseudomass. Flow might be preserved in all or part of the redundant cord because of inflowing arterial flow above the twist and obstructed venous flow beneath the twist. A partially twisted cord can have flow throughout its extent and might even look hyperemic (Fig. 4) [9].

#### Horizontal or altered lie

**Fig. 5:**
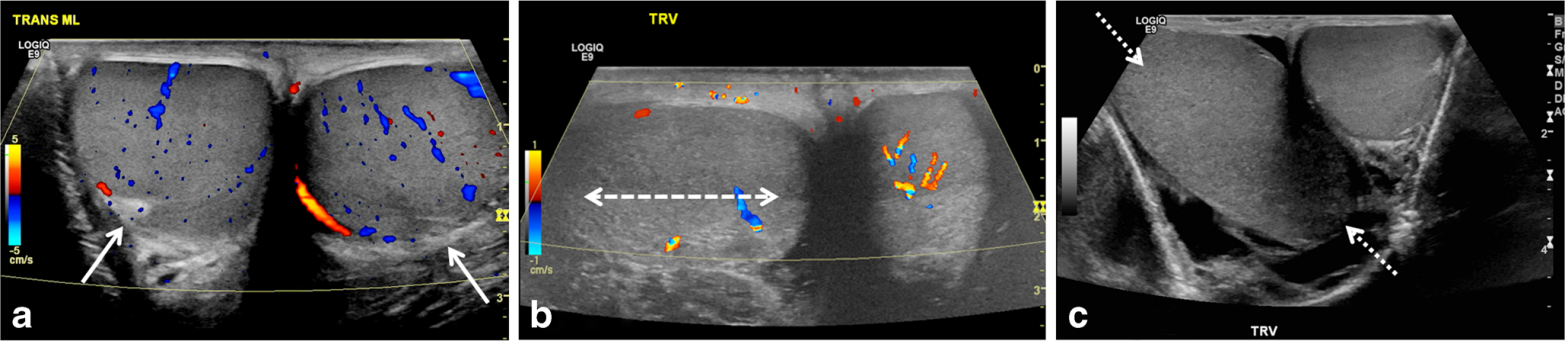
Testicular lie — normal and abnormal. **a** Color Doppler transverse US image of the testes in a 12-year-old boy with mild groin pain demonstrates normal vertical lie with the testes seen in round cross-sections and with the mediastinum testis (*arrows*) directed posterolaterally. **b** Color Doppler transverse US image of both testes in a 14-year-old boy who woke with acute right scrotal pain demonstrates abnormal horizontal lie of the right testis (*arrow*) with slightly decreased intratesticular flow compared to the normal left side. He had experienced similar episodes of pain in the past and was diagnosed with intermittent torsion. During orchiopexy 12 h later, a bell clapper deformity was noted bilaterally. **c** Gray-scale transverse US image of both testes in a 16-year-old boy with right testicular pain demonstrates abnormal oblique lie of the right testis (*arrows*), which is oriented diagonally compared to the normal left side. Intermittent torsion was diagnosed intraoperatively

Normally the testes lie in a vertical orientation. A horizontal lie is thought to result from abnormal attachments of the tunica vaginalis, namely the bell clapper anomaly [10]. Horizontal or altered/oblique lie has been known to be associated with intermittent torsion [11]. A transverse image of the normal testes side-by-side shows two round-cut sections through the mid-testis with the echogenic mediastinum testis directed posterolaterally on either side (Fig. 5). In case of horizontal lie, the affected testis is oriented horizontally or mediolaterally such that on the transverse mid-testis image, the cross sections are asymmetrical with the horizontal testis seen in long axis and the normal testis seen in short axis (Fig. 5) [6]. A testis in altered or oblique lie appears to be oriented diagonally (Fig. 5). Changing lie within the duration of the exam is a critical finding that suggests the testis is mobile within the tunica, though the testis can be mobile without being torsed. Similarly, an anteromedially directed mediastinum testis on the transverse image is a subtle but easily recognized sign of incomplete testicular fixation and might help in diagnosing intermittent testicular torsion.

### Sonographic features concerning for testicular torsion but also seen in other conditions

#### Globular testicular enlargement

**Fig. 6:**
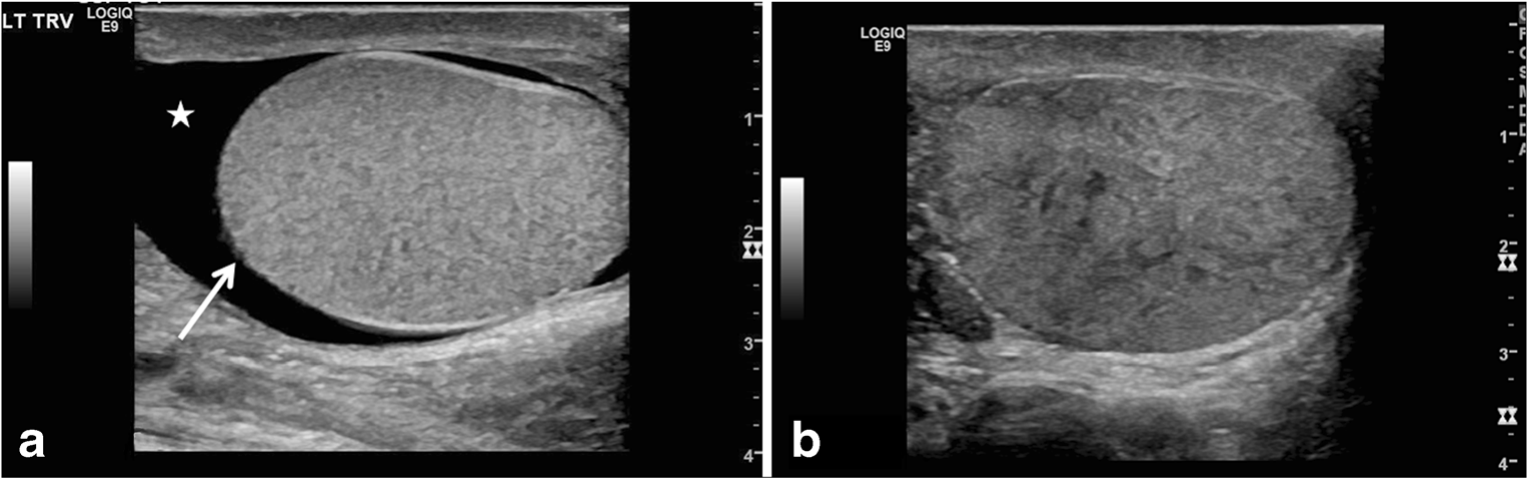
Globular testicular enlargement and heterogeneous echotexture. **a** Gray-scale transverse US image of the left scrotum in a 13-year-old boy with 3 days of intermittent left testicular pain shows globular shape of left testis with horizontal lie (*arrow*), decreased flow and redundant spermatic cord (neither shown), and reactive hydrocele (*star*). He was found to have a 180° twist on the left with abnormal epididymal attachment (the epididymis was attached only to the superior pole, suggesting bell clapper deformity) and he underwent bilateral orchiopexy after left detorsion. **b** Gray-scale longitudinal US image of the right testis in a 16-year-old boy with acute right testicular pain shows heterogeneous echotexture of the right testis; decreased flow and redundant spermatic cord were also present (not shown). Flow returned after detorsion and bilateral orchiopexy was performed. Intermittent torsion was diagnosed intraoperatively

A globular swollen testis resulting from vascular congestion is a worrisome feature for testicular torsion, especially in the absence of testicular hyperemia, and should not be mistaken for orchitis (Fig. 6). A rounder globular configuration is helpful even when testicular measurements are not discordant; this is sometimes related to measurement technique [8]. Testicular volumes are easier for comparison and are more accurate compared to the discrete size measurements as noted in our cases. A discrepancy in size, with the affected testis larger in volume than the asymptomatic side, is a key feature signaling the possibility of testicular torsion, similar to the sister sign for ovarian torsion.

#### Heterogeneous echotexture

Varying degrees of heterogeneous parenchymal echotexture might be seen in testicular torsion, and significant heterogeneity indicates late torsion and testicular nonviability (Fig. 6). Homogeneous echotexture portends extremely well for testicular viability [12]. Careful mapping of the testicular echotexture can lead to early diagnosis of potential segmental infarction and can also help during follow-up exam, when the echotexture might return to normal.

#### Epididymal enlargement without hyperemia

**Fig. 7:**
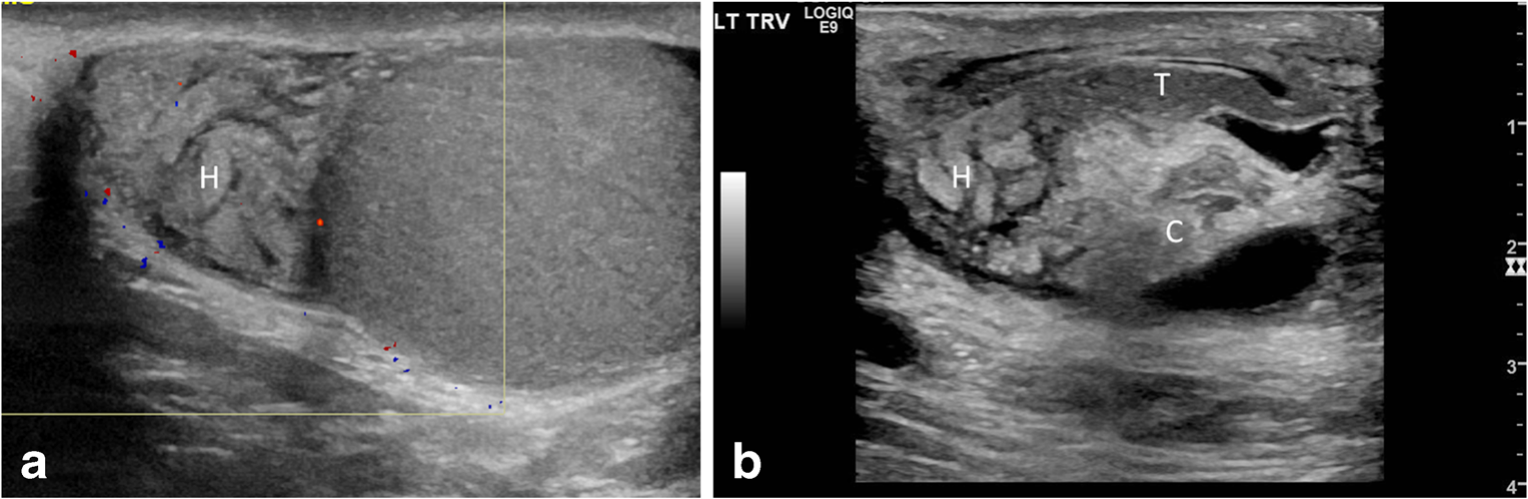
Epididymal enlargement without hyperemia. **a** Color Doppler longitudinal US image of the epididymal head and testis in a 12-year-old boy who woke with acute left testicular pain and nausea. Image shows an enlarged epididymal head (*H*) with a swollen lobular appearance and lack of intrinsic color flow. The intratesticular flow was decreased. He underwent emergent bilateral orchiopexy for a complete left torsion, and a bell clapper deformity was appreciated. Even though the epididymis was enlarged, it was avascular, thus ruling out epididymitis. **b** Gray-scale longitudinal US image of the epididymal-cord complex in a 7-year-old boy with 3 days of intermittent left testicular pain shows an edematous epididymal head (*H*) with a tangled echogenic cord (*C*) while the epididymal tail (*T*) appears relatively uninvolved. His left testis, however, showed somewhat decreased flow and he was diagnosed with partial torsion. A 360° twist was found during orchiopexy along with bilateral classic bell clapper deformity

The epididymis is inevitably involved in the torsion knot and it is often enlarged. The swollen epididymis looks like a heterogeneously echogenic and lobular structure containing radiating hypoechoic bands (Fig. 7). It can be impossible to tell epididymis from cord structures in the pseudomass. Hence we prefer the term “epididymal-cord complex” to better describe the conglomerate of edematous epididymis and convoluted spermatic cord (Fig. 7; Video 6, online supplementary material) [13]. The enlarged epididymis can show increased, decreased or normal flow. But a hypovascular or avascular enlarged epididymis in the setting of preserved testicular vascularity is highly worrisome for torsion [13]. An understanding of the epididymal-cord complex can preclude the erroneous diagnosis of an intrascrotal neoplasm.

#### Role of spectral Doppler in testicular torsion:

Intratesticular arteries characteristically have a low resistance pattern with a mean resistive index of 0.62 (range, 0.48–0.75) [3]. Asymmetrical dampened spectral waveform in the affected testis and absence or reversal of diastolic flow suggests testicular torsion in the proper setting [7]. Documenting Doppler waveforms and calculating resistive indices on both the affected and non-affected sides can be useful in determining differences in testicular perfusion.

### Torsion versus Epididymitis: Approach to diagnostic challenges

The clinical features considered predictive for testicular torsion include pain <24 h, nausea/vomiting, abnormal cremasteric reflex, and high position of the testis [14]. The best predictors for epididymitis include dysuria, a painful epididymis on palpation, altered epididymal echogenicity and increased peritesticular perfusion found on ultrasound exam [15]. Torsion of the appendix testis can be identified by the presence of the blue dot sign, a palpable nodule on the upper pole of the testis, or visualization of a relatively avascular nodule on ultrasound [15].

**Fig. 8:**
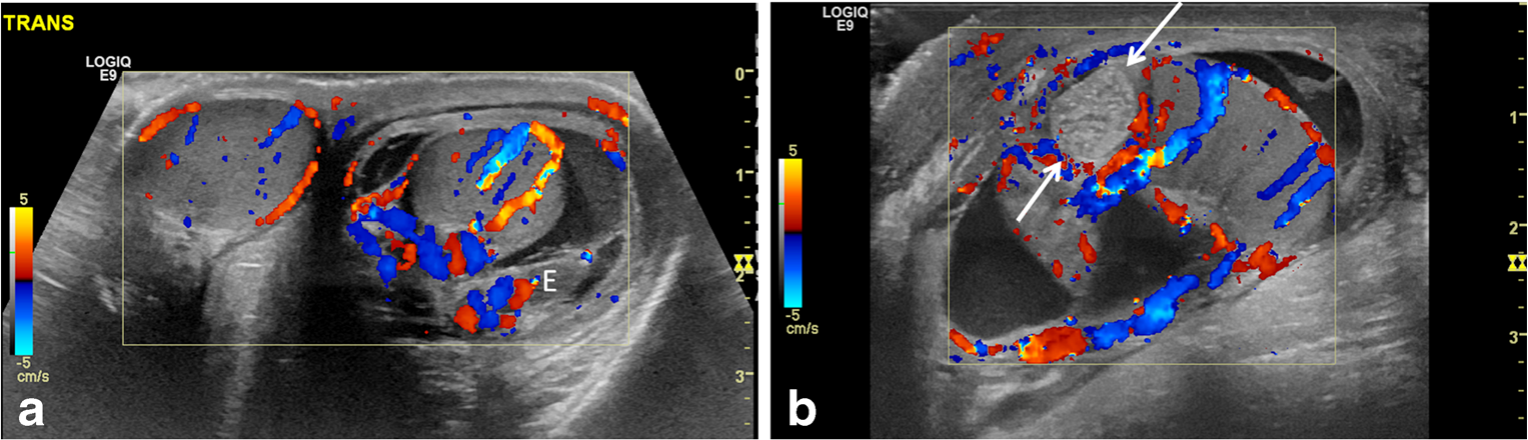
Epididymitis and testicular appendage torsion. **a** Transverse color Doppler US image of both testes in a 9-year-old boy with left-side pain shows hyperemia in the left testis and thickened epididymis (*E*) with surrounding hydrocele and left scrotal wall edema, all favoring left epididymo-orchitis. **b** Longitudinal color Doppler US image of the left scrotum in a 10-year-old boy with acute pain shows a globular, enlarged, echogenic and avascular nodular structure (*arrows*) at the superior pole of the testis, compatible with torsion of the testicular appendage. Not infrequently, reactive inflammatory change involves the ipsilateral testis and epididymis

Epididymitis is inflammation of the epididymis (Fig. 8; Video 7, online supplementary material); it occurs more frequently among adolescents but also occurs in younger boys. Sexually transmitted infection is a potential etiology in the adolescent. A urinary tract infection is another potential etiology. In children, infectious epididymitis can result from viral infections or refluxed urine secondary to an underlying anatomical abnormality [10, 16]. Epididymitis is much less common than torsion in the pre-adolescent boys; when advanced, the epididymis adheres to the testis and can result in epididymo-orchitis (Video 8, online supplementary material) [16].

Our sonographic protocol for suspected testicular torsion is described (Table 1). Asking the child which side hurts before starting is recommended so that the color Doppler parameters can be optimized in the normal testis, and then the affected side can be interrogated using the same settings. Phrases such as “slightly increased or decreased flow” and “symmetrical or asymmetrical flow” are descriptive, and using them to describe the testicular vascularity is encouraged because they can be extremely helpful in depicting the parenchymal flow in the report. In a normal testis, color flow is seen as a mix of red and blue long linear robust channels representing intratesticular arterial and venous flow. Decreased flow is usually manifested as scattered blips of flow in vessels that appear rather diminutive and resemble dots and dashes. Increased flow is easier to appreciate by the increased number and fullness of vessels. If no appreciable difference can be detected by the viewer, the flow is considered symmetrical. The decision about color Doppler flow is subjective but is best accomplished by looking at both testes simultaneously in the transverse plane, in real time. We recommend obtaining a midline transverse color static cine clip of both testes side-by-side to show real-time intrinsic flow at the beginning of the exam when the testes have not yet been manipulated by the transducer.

**Table 1:** Sonographic protocol for suspected testicular torsion

1	Transducer - Use the linear high-frequency 6- to 15-MHz probe. The linear 9-MHz probe can be used to obtain greater depth. In infants, the hockey-stick probe can be used. Start the examination by asking where it hurts.
2	First obtain a midline gray-scale transverse view to document lie of both testes.
3	Take transverse static color Doppler image of both testes.
4	While holding the probe still (do not sweep) in midline transverse mid-testis position, take a 3-s color cine clip of both testes side-by-side to show real-time intrinsic flow.
5	Take gray-scale longitudinal images in central, medial and lateral aspect of each testis.
6	Take gray-scale transverse images at superior, mid and inferior portions of each testis.
7	Document testis volume on each side.
8	Start with asymptomatic side in order to optimize color Doppler parameters, and then move to the affected side. Perform color Doppler of each testis.
9	Color Doppler exam with pulsed Doppler tracing of arterial and venous flow (angle correct if possible, and use power Doppler if flow is difficult to document). Try to measure testicular arterial resistive index.
10	Take gray-scale and color images of the epididymis in longitudinal and transverse planes.
11	Take gray-scale and color images of the spermatic cord in longitudinal plane. Follow the cord meticulously in its entire extent in the sac and through the inguinal canal till the internal ring.
12	Take cine sweeps of each testis in longitudinal and transverse planes.
13	Take cine sweep of each spermatic cord in longitudinal plane.
14	In case of any pathology, save images as needed.

Swollen epididymis and testis with testicular flow that is only minimally decreased, normal, or increased in boys with incomplete or intermittent testicular torsion can mimic epididymo-orchitis. The most common cause of acute scrotal pain in children is torsion of appendix testis, which can also mimic epididymo-orchitis. Therefore it is important to evaluate for the presence of avascular nodule that might represent the torsed appendage (Fig. 8; videos 9, 10 and 11, online supplementary material). Any time a child presents with acute scrotal pain, and findings resembling epididymitis without findings of torsed appendage, testicular torsion should be excluded. Suspicion of intermittent or incomplete testicular torsion should be raised in a child with a history of recurrent epididymitis or epididymo-orchitis, or abrupt pain that resolved or decreased at the time of diagnosis.

**Fig. 9:**
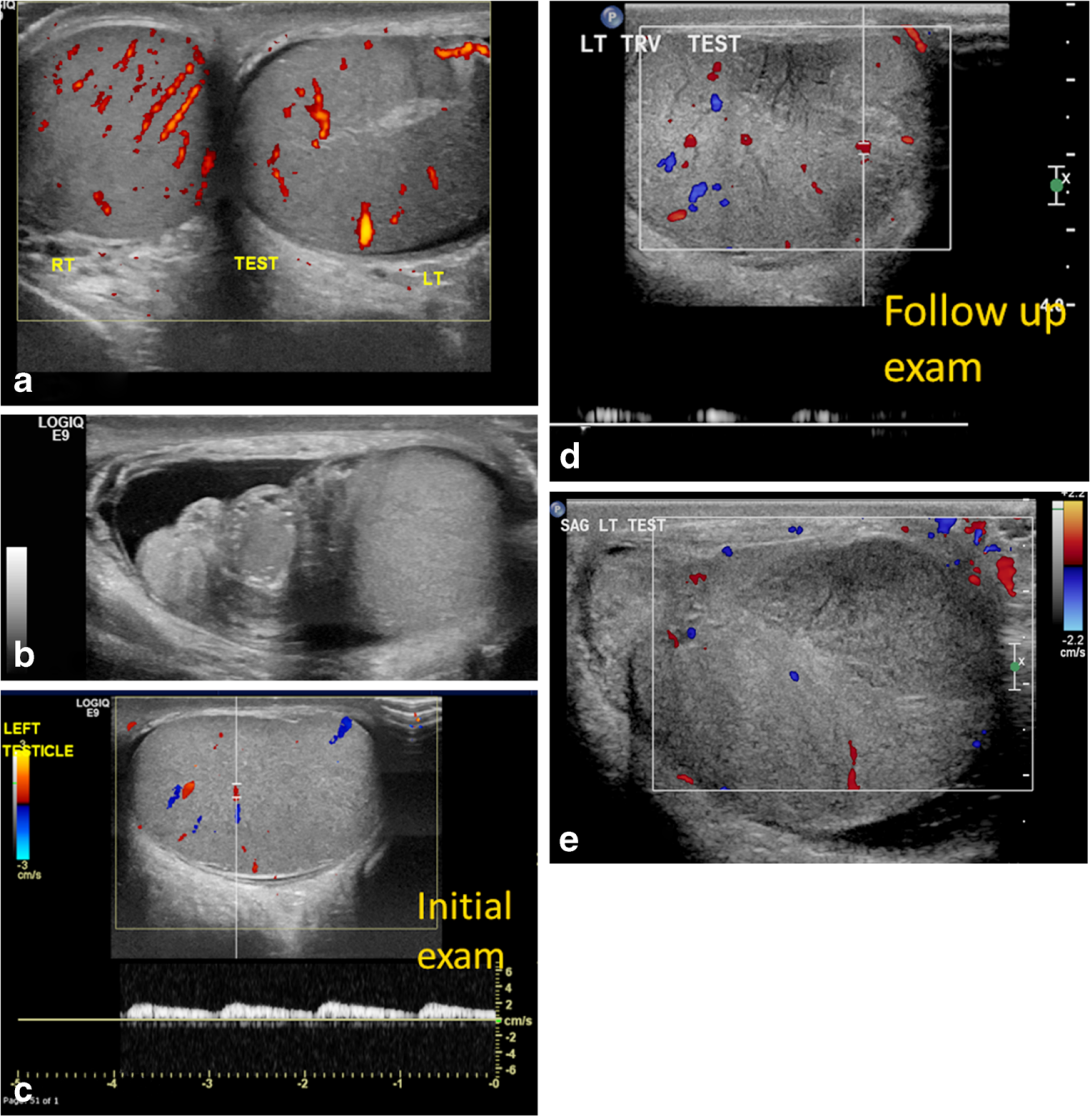
Partial torsion in a 13-year-old boy who presented with a 2-week history of intermittent left testicular pain and swelling. **a** Transverse power Doppler US image of both testes shows bilateral preserved flow with a slightly globular-appearing left testis. **b** Gray-scale longitudinal US image of the left testis and epididymis shows an enlarged left epididymal-cord complex and surrounding small hydrocele. The boy was discharged with a diagnosis of epididymitis because of the long confounding duration of symptoms and presence of intratesticular flow. **c** Color and pulsed Doppler US image of the left testis at initial presentation shows preserved intrinsic flow with normal arterial waveform. **d** Follow-up exam after 3 weeks demonstrates abnormally dampened left testicular flow and waveforms. **e** Color Doppler US image of left testis during at follow-up for persistent pain shows interval significantly enlarged left testis with decreased vascularity and a focal hypovascular parenchymal area concerning for infarct in the setting of incomplete torsion. He underwent left detorsion with bilateral orchiopexy

**Fig. 10:**
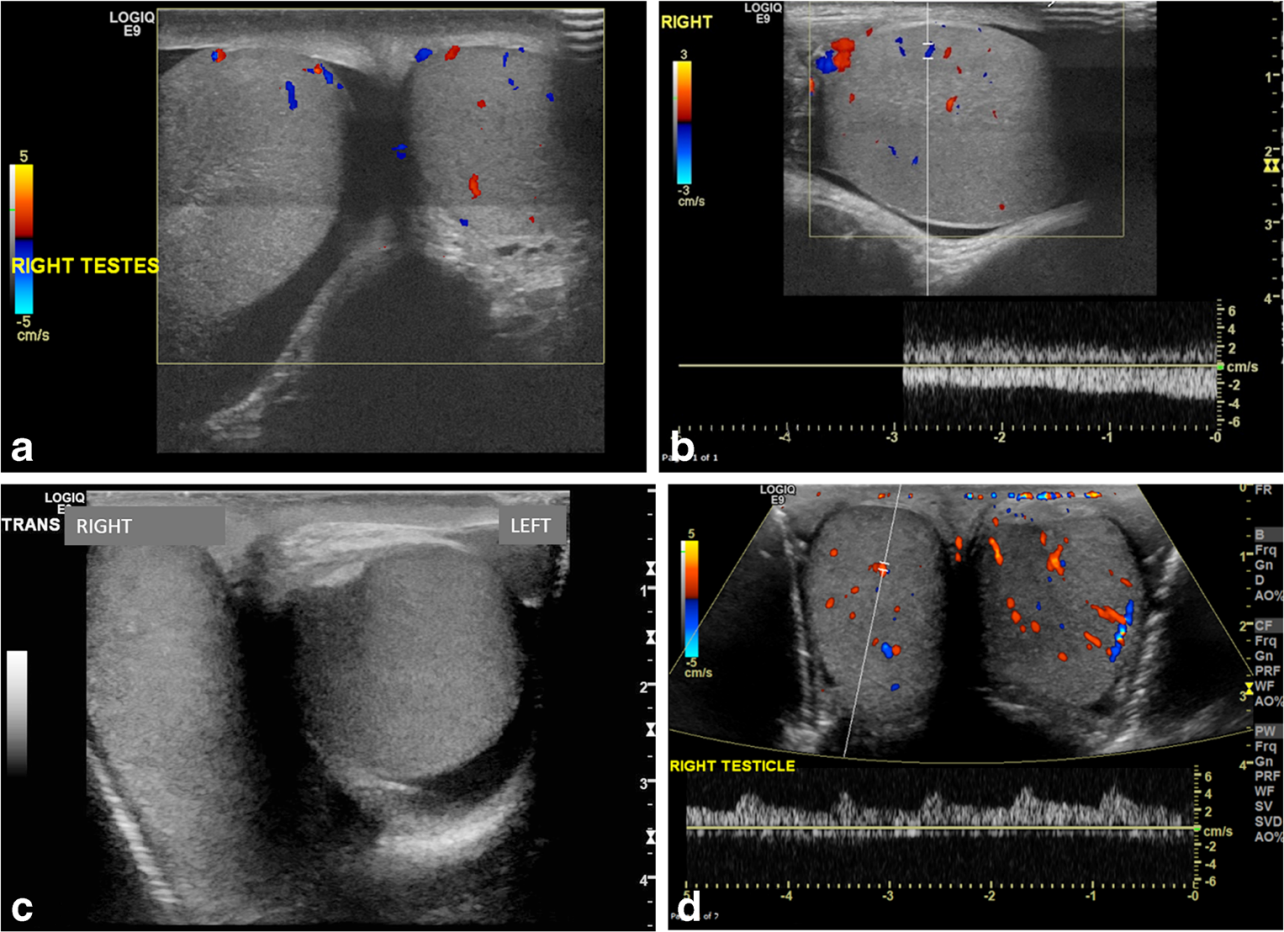
Complete torsion with preserved flow. **a** Transverse color Doppler US image of both testes in a 13-year-old boy with 8 h of right-side acute pain shows oblique lie and globular enlargement of the right testis with preserved flow and surrounding small reactive hydrocele. There is a linear transducer artifact at mid-testis level. **b** Longitudinal color Doppler US image of the right testis shows a globular testis with intratesticular flow. During surgery, complete torsion was diagnosed with a 360° twist and bilateral bell clapper deformity. **c** Transverse gray-scale image of both testes in a 15-year-old boy with acute right testicular pain shows horizontal lie of the right testis. The asymptomatic left testis incidentally demonstrates a medially directed mediastinum testis and trace fluid in the tunica vaginalis. **d** Transverse color Doppler US image demonstrates preserved intratesticular flow thought to be symmetrical. Complete torsion of the right testis was diagnosed at surgery, and bilateral orchiopexy was performed post detorsion

**Fig. 11:**
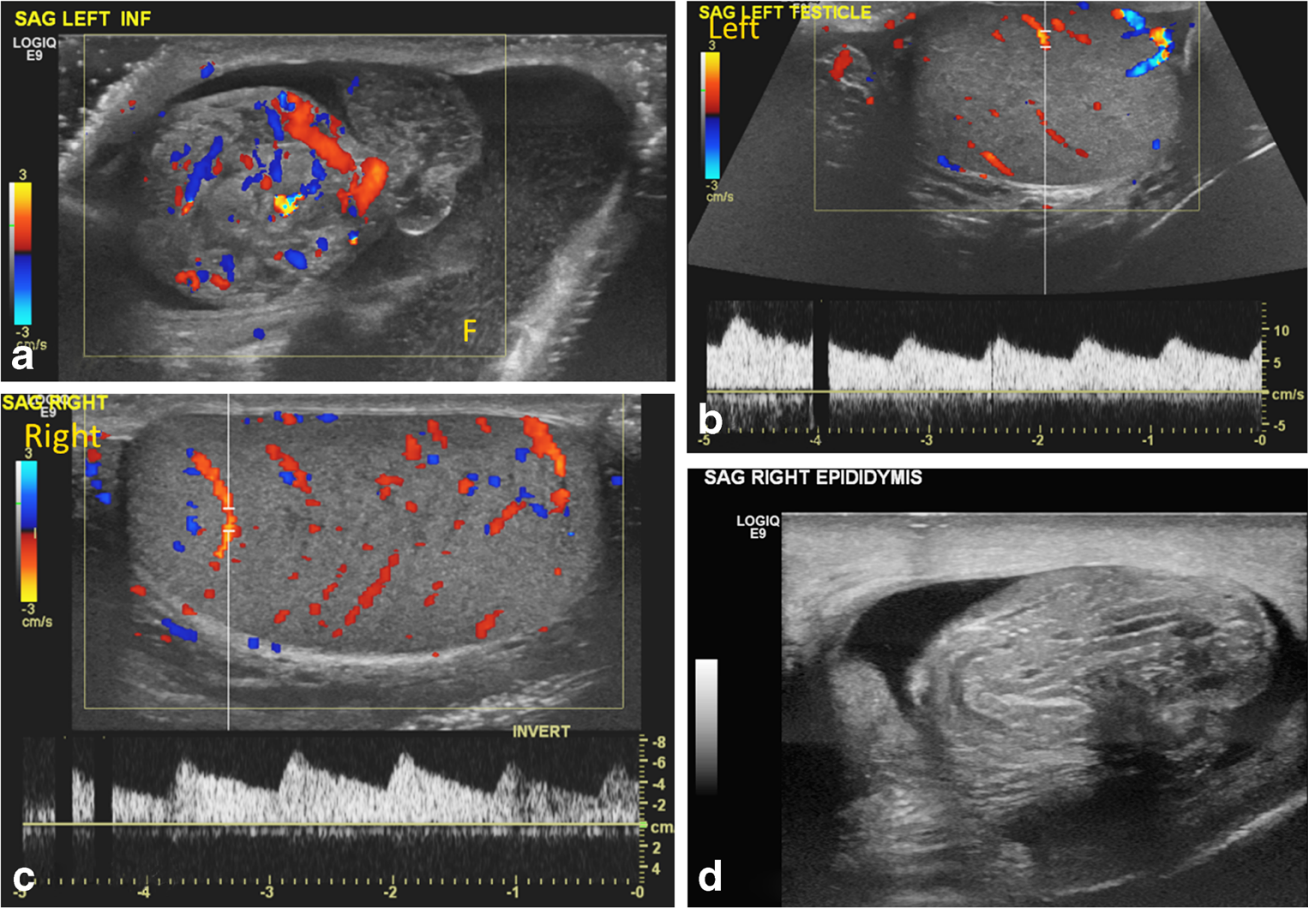
Bilateral partial torsion in a 15-year-old boy who presented with acute left testicular pain. **a** Color Doppler US image of the upper left scrotal sac shows an enlarged epididymal-cord complex with flow within, and surrounding complex hydrocele with mobile echoes. **b, c** Preserved slightly asymmetrical — left (**b**) less than right (**c**) — intratesticular flow and symmetrical waveforms are appreciated. The enlarged epididymal-cord complex was confused for epididymitis. **d** Follow-up exam after 4 months for recurrent testicular pain, now on the right side, shows an enlarged epididymal-cord complex in the right scrotum. Bilateral partial torsion (270° twist on the right side and 90° twist on the left side) and bilateral bell clapper anomaly were detected at orchiopexy

Cases of complete torsion with absent flow in affected testis are usually diagnosed without difficulty by sonography [17]. In late-phase torsion (greater than 24 h), in addition to lack of color flow within the testis, a “halo sign” consisting of a rim of increased pudendal flow surrounding the testis is present [13]. In both situations, the epididymis is also frequently enlarged and avascular [13]. Epididymitis is the most common entity confused with testicular torsion, and a study of relevant malpractice claims found that in 61% of these cases, torsion was misdiagnosed as epididymitis [18]. In case of partial or intermittent torsion with recurrent episodes of testicular pain, sonographic examinations can be difficult and ambiguous: testicular flow may still be present but decreased in comparison to the contralateral side or flow may be apparently symmetrical with the contralateral side (Fig. 9). If the flow is markedly decreased compared to the asymptomatic side, testicular torsion is a more likely diagnosis. If the decrease in flow is subtle, a conclusive diagnosis from sonography is more difficult. In cases with complete torsion and near-symmetrical flow, a high degree of suspicion is necessary for accurate diagnosis (Fig. 10). In addition, flow might be maintained within the epididymis, which is often enlarged and therefore not infrequently results in an erroneous diagnosis of epididymitis. If spontaneous detorsion has occurred, the symptoms resolve and the resultant hyperemia and enlargement of the epididymis and testis can result in the erroneous diagnosis of epididymo-orchitis. Systematic high-resolution sonography of the cord can significantly influence surgical management [19]. The sonographic findings depend not only upon the degree of spermatic cord rotation and the length of time of the torsion, but also upon how tightly the cord is twisted (Fig. 11) [20].

Incomplete/partial testicular torsion is a difficult diagnosis both clinically and sonographically. Arce et al. [21] showed that rotated cord structures were visualized with gray-scale ultrasound in all six of their patients with surgically proven testicular torsion, despite the presence of intratesticular color flow, suggesting a diagnosis of incomplete testicular torsion. The torsion knot has been reported on MR imaging in an adult with 6 weeks of symptoms [22]. Both testicular MRI and contrast-enhanced sonography in the future might be a useful complement to clinical and baseline imaging in difficult/sonographically equivocal cases, although there are no prospective studies in the pediatric population [23]. In a retrospective study by Galina et al. [9], the epididymis was always sonographically abnormal in boys with testicular torsion, being minimally or grossly displaced in relation to the testis and spermatic cord.

Clinical correlation is essential. Severe nausea and vomiting and abrupt onset of pain favor torsion. A more common clinical mimic of testicular torsion is an acutely torsed testicular appendage, which is treated conservatively [3]. Presence of subcutaneous superficial soft-tissue swelling favors inflammatory etiology, either epididymitis or appendage torsion, rather than testicular torsion, in which case the subcutaneous edema is typically seen later in the presentation. Scrotal exploration should be considered whenever there is suspicion for testicular torsion, even if the ultrasound findings are not specific.

## Testicular salvage

Likelihood of salvage of the testis is directly related to the time between symptom onset and detorsion [24]. However in our experience salvage is unpredictable depending on how tightly or loosely the cord was twisted, and hence surgery should not be delayed after the diagnosis of torsion is established, even if the time to presentation exceeds the 6- to 10-h window. A testis might become nonviable as early as 4 h after a 720° twist, or it might remain viable for several days if the torsion is incomplete. In one of our cases, salvage was achieved after 3 weeks from initial diagnosis of epididymitis to final diagnosis of incomplete torsion (Fig. 9). Anatomical variability might account for differences in the duration of viability of the torsed testis [25]. Initially after salvage, the testes sometimes appear in good condition; however long-term follow-up of salvaged testes post orchiopexy can be helpful in determining the effects of torsion. Manual detorsion of the testis can restore blood flow while the boy awaits surgical correction. In a study of 133 patients with testicular torsion, successful manual detorsion was associated with a salvage rate of 97% compared with 75% salvage in patients in whom detorsion was not attempted or not successful [26].

## Conclusion

Testicular gray-scale ultrasound is the modality of choice for the imaging evaluation of acute scrotal pain. The presence of intratesticular flow does not exclude testicular torsion. Testicular vascularity can look symmetrical with the contralateral asymptomatic side with preserved arterial and venous flow and still represent testicular torsion. An integral part of the exam is to look for an abrupt change in configuration of cord at the external inguinal ring or in the scrotal sac. The presence of redundant spermatic cord within the scrotum is highly suspicious for testicular torsion. An enlarged epididymal-cord complex representing the torsion knot/pseudomass is more frequently identified at the sonographic examination compared to the more classic whirlpool sign of twisted spermatic cord. Residual flow might be preserved in parts of the cord when the twist is not tight enough to completely obliterate the flow. An astute analysis of the cord and lie of the testis can prevent the overdiagnosis of epididymitis. We encourage the use of simultaneous cine clips of testes side-by-side at the start of the exam to document lie and assess inherent flow. A group discussion among the radiologist, emergency room physician and urologist is recommended for proper management; manual detorsion of the testis might be considered to improve chances of salvage while the patient awaits surgery.

## Electronic supplementary material

Video 1 (AVI 2110 kb)

Video 2 (AVI 4892 kb)

Video 3 (AVI 9569 kb)

Video 4 (AVI 4047 kb)

Video 5 (AVI 5098 kb)

Video 6 (AVI 5497 kb)

Video 7 (AVI 1216 kb)

Video 8 (AVI 2159 kb)

Video 9 (AVI 3195 kb)

Video 10 (AVI 4450 kb)

Video 11 (AVI 1455 kb)
